# Components and Pharmacodynamical Mechanism of Yinfupian Based on Liquid Chromatography-Mass Spectrometry and Proteomics Analyses

**DOI:** 10.3389/fphar.2021.680640

**Published:** 2021-06-28

**Authors:** Heng-li Tong, Hao Chen, Fei-peng Gong, Ling-yun Zhong, Jing Zhu, Song-hong Yang

**Affiliations:** ^1^ Laboratory of Traditional Chinese Medicine Preparation, School of Pharmacy, Jiangxi University of Traditional Chinese Medicine, Nanchang, China; ^2^ Department of Orthopedic, Jiangxi Provincial People’s Hospital, Nanchang, China

**Keywords:** Aconitum, preparation, chemical components, pharmacology, proteomics, yang deficiency

## Abstract

**Objective:** According to the treatment records of Yang deficiency syndrome (YDS) with characteristic decoction pieces of lateral root of *Aconitum carmichaelii*—Yinfupian (YF) in traditional Chinese medicine prepare school, known as “Jianchangbang”. The aim of this study was to investigate differences in the composition and therapeutic mechanism of the unprocessed lateral root of *Aconitum carmichaelii* (ULRA) and its processed product (YF).

**Methods:** Ultra-performance liquid chromatography-quadrupole time-of-flight mass spectrometry and orthogonal partial least squares discriminant analysis method were used to determine and screen the main components of ULRA and YF. Changes in the histological structure and morphology of gonads in rats were observed using hematoxylin-eosin. Enzyme-linked immunosorbent assay was used to determine the contents of serum cyclic adenosine monophosphate and cyclic guanosine monophosphate in YDS rats treated with ULRA and YF. Tandem mass tag proteomics analysis was used to identify the differentially expressed proteins in YDS rats treated with ULRA and YF.

**Results:** Both ULRA and YF exerted certain therapeutic effects on rats with YDS. They improved the gonadal morphology and increased the contents of serum cyclic adenosine monophosphate and cyclic guanosine monophosphate. After processing of ULRA into YF, the content of C19-diester-diterpenoid alkaloids decreased (converted into C19-monoester-diterpenoid alkaloids and C19-alkylol amine-diterpenoid alkaloids), whereas that of C20-diterpene alkaloids increased. Proteomics analysis showed that cytochrome P450 and aldehyde oxidase 3 (AOX3) were downregulated, whereas cathepsin G (CTSG) was upregulated in rats with YDS. Treatment with ULRA mainly downregulated the expression of α-actinin, fast skeletal troponin, creatine kinase, and myosin. Treatment with YF mainly upregulated the expression of mitochondrial ribosomal protein and mitochondrial inner membrane protein.

**Conclusion:** ULRA and YF exerted good therapeutic effects on YDS; the main difference in components between these preparations was in C19-diterpenoid alkaloids. ULRA mainly acts on the muscle contraction-related proteins and is closely related to inflammation and myocardial injury. YF mainly acts on the mitochondrial proteins and is closely related to adenosine triphosphate energy metabolism.

## Introduction

Yang deficiency syndrome (YDS) is a physical state characterized by chilly sensation (i.e., cold limbs) and dysfunction of the viscera. The main clinical manifestations are “dread cold, coolness of extremities, fatigued body and lack of strength, depression, hypomnesis, clear and large amount of urine” (Shi et al., 2018). This condition is associated with heart failure, hypothyroidism, chronic gastritis, primary nephrotic syndrome, infertility, and other diseases (Xia et al., 2010; Yang et al., 2010; Zou et al., 2011; Fan, 2014; Weng, 2014). Physiological, biochemical, and genomic studies on YDS (Zhang et al., 2019) have shown that YDS is associated with hypofunction of the hypothalamic-pituitary-adrenal, hypothalamic-pituitary-thyroid, and hypothalamic-pituitary-gonad axes, as well as disturbance of the cyclic nucleotide system and immune function. Polymorphisms of susceptibility genes in patients with YDS have been linked to the levels of cyclic adenosine monophosphate (c-AMP) and cyclic guanosine monophosphate (c-GMP), memory, metabolic energy status, and immune function (Yao et al., 2015).

*Aconitum* had been used medicinally and as a poison in both Western and Eastern countries for centuries. It is currently used medicinally in different parts of the world, such as China and Solčavsko (Slovenia) (Zhou et al., 2014; Povšnar et al., 2017). The lateral root of *Aconitum carmichaelii* (LRA) is termed “Fuzi” in traditional Chinese medicine; it is also called “Bushi” and “Kyeong-PoBuja” in Japan and Korea, respectively. It has shown efficacy in reviving the Yang for resuscitation, tonifying fire, and helping Yang, as well as dispersing cold and relieving pain (Chinese Pharmacopoeia Commission, 2020). The application of LRA has a long history, with remarkable therapeutic effects. The primary active component of *Aconitum* is alkaloid, mainly used to treat rheumatoid arthritis and cardiovascular diseases (Zhou et al., 2014). The diterpenoid alkaloids of LRA can be divided into diester, monoester, and alkylol amine alkaloids, according to the presence or absence of an ester bond at the C4 and C8 positions (Hu et al., 2010). Alkylol amine alkaloids and other water-soluble nitrogen-containing organic compounds are collectively referred to as water-soluble alkaloids. It has been reported that higenamine (Kosuge and Yokota, 1976), coryneine chloride (Konno et al., 1979), salsolinol (Cheng and Liang, 1982), uracil (Nizhenkovska, 2015), and fuzinoside (Li et al., 2015) are the cardiotonic active components of LRA. In recent years, further studies have shown that the water-soluble diterpenoid alkaloids found in LRA, including beiwutinine, mesaconine, karakoline, aconine, isotalatizidine, hypaconine, and 3-deoxyaconine, are cardiotonic active components (He et al., 2014a; He et al., 2014b; Wang et al., 2014). Fuziline, neoline (Xiong et al., 2012), talatisamine (Chen et al., 2014), and neoline also have significant analgesic effect (Suzuki et al., 2016). Songorine exerts anti-inflammatory, analgesic, and anti-anxiety effects (Yu et al., 2014; Khan et al., 2018). Fuziline has demonstrated efficacy against shock and improves myocardial ischemia (Gong et al., 2016). In addition, the water-soluble alkaloids found in LRA have anti-arrhythmic and anti-hypertensive effects (Fujita et al., 2009; Li et al., 2014). Therefore, this plant has broad prospects for the development of new drugs and applications. For centuries, Fuzi has been effectively used in treating various diseases associated with YDS. Jianchangbang is an ancient school of medicine in the south of China. Its method of preparing LRA through steaming with ginger juice is unique and famous. It is well established that Yinfupian (YF) has high efficacy and low toxicity, and is suitable for the treatment of YDS in clinical practice. At present, the impact of the components of *Aconitum* on proteins related to YDS remains unclear. The aim of this study was to investigate changes in the components of LRA before and after preparation, content of serum cyclic nucleotides, gonadal tissue morphology, and the relationship between components and proteins in rats with YDS. The proteomic changes in liver tissue were detected using tandem mass tag (TMT) mass spectrometry (MS). Proteomics may provide new information for high-throughput analysis, and it has been used in investigating the pathogenesis of YDS with great coverage of the proteome (Huang and Liang, 2005; Lu et al., 2012; Li et al., 2017). These research studies provide new data for the therapeutic value of LRA and its processed products in diseases related to YDS, and promote the development of new potential biomarkers for abnormalities related to Yang deficiency.

## Materials and Methods

### Preparation of Medicinal Materials

Salted LRA (LRA soaked in bittern water; Batch No. 20180918) and ginger (fresh rhizomes of *Zingiber officinale Roscoe*) was purchased from the Chinese medicinal materials market of Zhangshu (China). All materials were authenticated by Professor Qianfeng Gong of Jiangxi University of Traditional Chinese Medicine (Nanchang, China). Unprocessed LRA (ULRA) was obtained by rinsing the salted LRA. ULRA was moistened with 15% fresh ginger juice (quality %) for 12 h and steamed for 8 h to obtain YF.

Administered solution: ULRA and YF were respectively soaked in water (×10 their volume) for 1 h, boiled for 30 min, and filtered. The obtained residues were soaked again in water (×6 their volume), boiled for 30 min, and filtered. The filtrates were combined and concentrated to 1.2 g/ml. The concentrated solution was stored at 4°C.

Standard solution: All standards (Weikeqi biotech Co., Ltd., Chengdu, China) were weighed and added isopropanol-dichloromethane (1:1) mixed solution to prepare solution contained 10.32 μg/ml aconitine (Batch NO. 130723), 37.38 μg/ml mesaconitine (Batch NO.130402), 5.75 μg/ml hypaconitine (Batch NO. 130509), 33.34 μg/ml benzoylaconine (Batch NO. 130516), 7.92 μg/ml benzoylmesaconine (Batch NO. 130505), and 5.38 μg/ml benzoylhypaconine (Batch NO. 130401).

Sample solution for HPLC: ULRA and YF were respectively soaked in water (×10 the volume) for 1 h, boiled for 30 min, and filtered. The obtained residues were soaked again in water (×6 the volume), boiled for 30 min, and filtered. The filtrates were combined, concentrated and dried into powers. The powders (2 g) of ULRA and YF decoction mixed with ammonia (3 ml), and immersed in isopropanol-ethyl acetate (1:1) mixed solution (50 ml) extracted under ultrasonic condition for 30 min, and then filtered. The filtrate (25 ml) evaporated to dryness under 40°C and low pressure condition. The obtained residue dissolved by isopropanol-dichloromethane (1:1) mixed solution (5 ml), and then filtrated through a filter membrane (0.22 μm).

Sample solution for UPLC-quadrupole-TOF-MS: The medicine (100 g of ULRA or YF) and 15 times the volume of water (×15 their volume) were placed into round bottom flasks, soaked for 30 min, subjected to heat reflux, decocted for 30 min, filtered and concentrated to 1 g/ml. Subsequently, 5.0 ml of the above concentrated solutions were transferred into 50 l volumetric flasks. Water was added to dissolve the concentrated solution. The supernatants were filtered using a 0.22-microporous membrane. The primary filtrates were discarded and the remaining filtrates were stored as sample solution.

### Conditions of Chromatography and Mass Spectrometry

An Ultimate 3000 high performance liquid chromatography (HPLC) (Dionex, Sunnyvale, CA, United States), equipped with a PDA-3000 diode array ultraviolet detector (Dionex, Sunnyvale, CA, United States) and a chromeleom workstation was used. Symmetry C18 column (5 μm, 4.6 mm × 250 mm, Waters Corporation, Milford, MA, United States) was used to determine the six alkaloids standards and the components of ULRA and YF.

**FIGURE 1:**
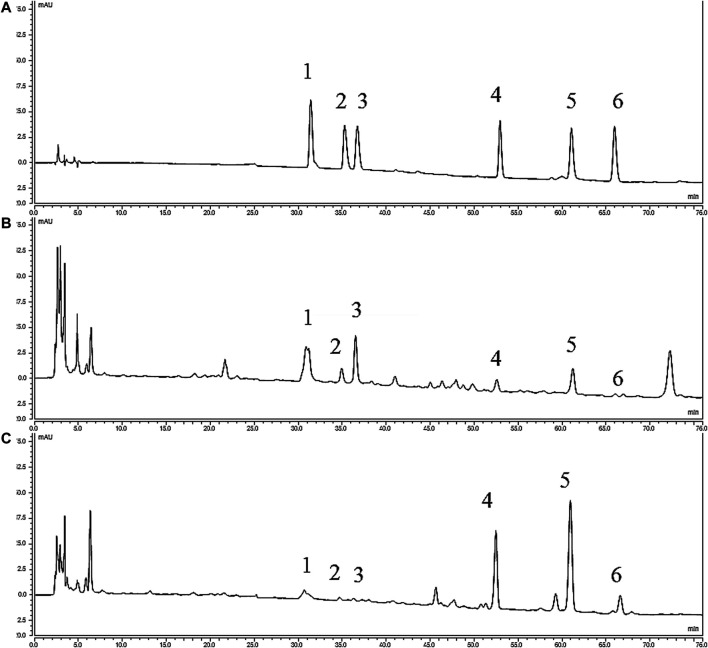
HPLC chromatograms of various alkaloids. **(A)** Standards; **(B)** YF; **(C)** ULRA; (1), Benzoylmesaconine; (2), Benzoylhypaconine; (3), Benzoylaconine; (4), Mesaconitine; (5), Aconitine; (6), Hypaconitine; HPLC, high performance liquid chromatography; ULRA, unprocessed lateral root of *Aconitum carmichaelii*; YF, Yinfupian.

The following gradient system was used with 100% acetonitrile (solvent A) and 0.04 mol/L Ammonium acetate aqueous solution (PH = 10, Adjusted by ammonia water) (solvent B): 0–8 min: 18% of A; 8–35 min: linear 18–35% of A; 35–45 min: linear 35–45% of A; 45–55 min: linear 45–50% of A; 55–65 min: linear 50–55% of A; 65–75 min: 55% of A. The flowing rate was 1.0 ml/min, the detection wavelength was at 235 nm, the column temperature was at 35°C, and the injection volumn was 20 μl. The chromatograms are shown in Figure 1.

An ultra-high performance liquid chromatography (UPLC) tandem four-stage rod time-of-flight (TOF) mass spectrometer (Triple TOF 5600+; AB Sciex), equipped with an Analyst1.6 Chromatographic workstation and MS analysis software (PeakView v1.2; AB Sciex), was used. Nexera UPLC LC-30 (Shimadzu Corporation, Kyoto, Japan) and ACQUITY UPLC® BEH C18 column (1.7 μm, 2.1 mm × 100 mm; Waters Corporation, Milford, MA, United States) were used to determine the components of ULRA and YF.

The gradient system was 0.1% formic acid aqueous solutions (solvent A)-100% acetonitrile (solvent B): 0–25 min, 95–60% A; 25–32 min, 60–5% A; 32–35 min, 5–95% A; Experiment: Positive Ion Mode, Electron Spray Ionization, ionization temperature: 500 °C, curtain gas: 40 psi, ion source gas1: 50 psi, ion source gas2: 50 psi, ion spray voltage: 5,500 eV, collision energy: 40 eV, declustering potential: 100 eV, mass scan range: *m*/*z* 100–1,000. The flow rate was 0.5 L/min, the column temperature was 40°C, and the injection volume was 2 μl.

### Calibration of Six Alkaloids and Method Evaluation

Standard samples of alkaloids were prepared into appropriate concentration, and the calibration curve for each alkaloid was performed with six quantities by plotting the peak area vs. the concentrations of the alkaloids. The results of all calibration curves are shown in Table 1.

**TABLE 1:** Calibration curves of various alkaloids.

Alkaloids	Regression equations	*R* ^2^
Aconitine	Y = 0.2050X+0.3294	0.9994
Mesaconitine	Y = 0.3915X+0.4621	0.9992
Hypaconitine	Y = 0.4043X+0.8154	0.9996
Benzoylaconine	Y = 0.6271X+0.3214	0.9992
Benzoylmesaconine	Y = 0.7897X+0.7180	0.9996
Benzoylhypaconine	Y = 0.3405X+0.1127	0.9993

Precision and stability was calculated within 24 h (*n* = 6) with the standard solution of the six alkaloids. Repeatability and average recovery was tested by sample solutions of ULRA and standard solutions of the six alkaloids, and they were injected six times. All indexes of method evaluation evaluated by calculating the relative standard deviation (RSD) (Table 2).

**TABLE 2:** RSD of method evaluation for various alkaloids (*n* = 6).

Alkaloids	Precision (%)	Stability (%)	Repeatability (%)	Average recovery (%)
Aconitine	1.08	0.38	0.35	0.16
Mesaconitine	0.75	0.52	0.43	0.63
Hypaconitine	0.25	0.73	0.86	1.40
Benzoylaconine	0.65	0.46	0.74	0.59
Benzoylmesaconine	0.54	0.23	0.65	0.30
Benzoylhypaconine	1.02	0.35	0.47	1.30

### Data Collection and Analysis of Ultra-High Performance Liquid Chromatography-Quadrupole-Time-of-Flight-Mass Spectrometry

**FIGURE 2:**
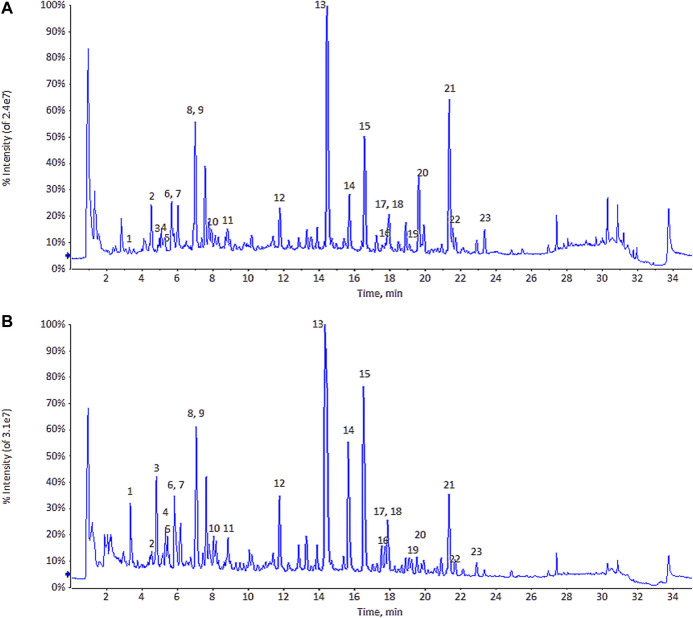
The total ion chromatogram (TIC) of ULRA **(A)** and YF **(B)**. ULRA, unprocessed lateral root of *Aconitum carmichaelii*; YF, Yinfupian.

The original data were imported into MarkView v1.2.1 and PeakView v1.2 software (both from AB Sciex) to determine the retention time, mass-to-charge ratio, intensity data, and information of two-stage MS (MS2). The total ion chromatogram of ULRA and YF is shown in Figure 2. Subsequently, the data were imported into Simca-p 14.1 software. To observe the contour differences between groups and more effectively identify differences in markers, orthogonal partial least squares discriminant analysis (OPLS-DA) was performed. This analysis yielded the OPLS-DA score chart and similarity-plot (S-plot) diagram, which could directly reflect the contribution rate of differences between groups. The markers with variable important in projection (VIP) > 1 and *p*-values <0.05 were considered potentially highly related to differences in the decoction pieces. The qualitative analysis of these markers was performed by comparing evidence form the literature and data retrieved from databases.

### Experimental Animal Treatments and Sample Collection

The animal study was approved by the Ethics Committee on Laboratory Animals of Jiangxi University of Traditional Chinese Medicine. Sprague–Dawley rats (24 males and 24 females, each weighing 200 ± 20 g; laboratory animal license no. 3700920018,748) were purchased from Jinan Pengyue Experimental Animal Breeding Co., Ltd. (Jinan, China) (license key number: SCXK (Lu) 2014 0007). All rats after acclimatizing in normal atmospheric temperature for 5 days and divided randomly into four groups containing equal numbers of male and female animals, namely the blank (B) group (*n* = 12), model (M) group (*n* = 12), ULRA group (*n* = 12), and YF group (*n* = 12).

Rats in the model and treatment groups received *Rheum officinale* (dried roots and rhizomes of *Rheum officinale* Baill) powder 1.0 g and goldthread (dried rhizomes of *Coptis chinensis* Franch) powder 1.0 g (namely, 2 g of herb powder/8 ml water suspension, 1 ml/100 g body weight, once daily for 7 days, at the same time) through oral gavage. After the administration, the rats were subjected to exhaustive swimming once daily to establish the model of YDS. Rats in the blank group received the same amount of distilled water daily through oral gavage. On day 8, rats in the treatment groups received ULRA and YF concentrated solution at 12 g (crude medicine)/kg (bodyweight) per day. Rats in the blank and model groups received the same amount of distilled water by oral gavage for 7 days.

Blood samples were collected from the abdominal aorta after anesthesia using 5% pentobarbital sodium (5 mg/100 g bodyweight, intraperitoneally). Next, the liver tissues were rapidly excised, frozen in liquid nitrogen, and stored at −80°C for protein extraction. Subsequently, testicular and ovarian tissues were excised, sectioned, embedded in paraffin, and stained with hematoxylin-eosin. Serum was separated by centrifugation at 3,000 rpm for 5 min at 4°C after standing for 15 min to detect c-Amp and c-GMP using enzyme-linked immunosorbent assay kits (LOT: 20191108; Nanjing Jiancheng Bioengineering Institute, Nanjing, China). Throughout the experimental period, there was no occurrence of death in the animals. At the end of the experiment, euthanasia was performed under anesthesia with sodium pentobarbital. The data are expressed as the mean ± standard deviation, and comparison between groups was performed using one-way analysis of variance.

### Tandem Mass Tag-Based Proteomics

Liver tissues were added to SDT lysis buffer (4% sodium dodecyl sulfate [SDS; 161-0302; Bio-Rad, Hercules, CA, United States]; 100 mM Tris-HCl [A6141; Sigma, Saint Louis, MO, United States]; 1 mM dithiothreitol [DTT; 161-0404; Bio-Rad, Hercules, CA, United States]; pH 7.6), transferred into a 2-ml centrifuge tube with an appropriate amount of quartz sand (MP 6910-050; MP Biomedicals, Santa Ana, CA, United States) and 1/4-inch ceramic bead (MP 6540-034; MP Biomedicals, Santa Ana, CA, United States), and crushed into homogenate (24 × 2, 6.0 M/S, 60 s, twice) using a MP fastprep-24 homogenizer (MP Biomedicals). Samples were subjected to ultrasound with an ultrasonic crusher (JY92-II; Scientz, Ningbo, China) (power: 80 W, duration: 10 s, intervals: 15 s, repetitions: 10) and placed in boiling water for 15 min. The homogenate was centrifuged at 14,000×*g* for 40 min, the supernatant was filtered with a 0.22-μm membrane, and the filtrate was collected. The bicinchoninic acid (BCA) method (BCA quantitative Kit; P0012; Beyotime, Nanjing, China) was used for protein quantification. The samples were stored at −80°C.

Protein from each group (20 μg) was added to 5× buffer solution (10% SDS, 0.5% bromophenol blue, 50% glycerin, 500 mM DTT, 250 mM Tris-HCL, pH 6.8) and placed in boiling water for 5 min. Next, 12.5% SDS-polyacrylamide gel electrophoresis (constant current, 14 Ma, 90 min; eps601; GE Healthcare, Marlborough, MA, United States) and Coomassie brilliant blue staining were performed.

Protein solutions (30 μl) were obtained from each group. DTT was added to reach a final concentration of 100 mM. Subsequently, the solutions were placed in boiling water for 5 min and cooled to room temperature. Uric acid (UA) buffer (200 μl; 8 M urea [161-0731 Bio-Rad]; 150 mM Tris-HCl; pH 8.0) was added, mixed well, transferred into a 10 kDa ultrafiltration centrifuge tube (Sartorius, Gottingen, Germany), and centrifuged at 14,000×*g* for 15 min; the filtrate was discarded (this step was repeated once). Iodoacetamide buffer (100 μl; 100 mM iodoacetamide [163-2109; Bio-Rad] in UA) was added, oscillated at 600 rpm for 1 min, reacted in the dark at room temperature for 30 min, and centrifuged at 14,000 × *g* for 15 min. UA buffer (100 μl) was added and centrifuged at 14,000 × *g* for 15 min; this step was repeated twice. Subsequently, 100 mM triethylamonium bicarbonate (TEAB) buffer (100 μl) was added, and the mixture was centrifuged at 14,000 × *g* for 15 min; this step was repeated twice. Trypsin buffer (40 μl; 4 μg trypsin [317107; Promega coporation, Madison, WI, United States) in 40 μl 100 mM TEAB buffer) was added, oscillated at 600 rpm for 1 min, and placed at 37°C for 16–18 h. The collection tubes were replaced, the samples were centrifuged at 14,000 × *g* for 15 min; ×10 diluted 100 mM TEAB buffer (40 μl) was added, and the samples were centrifuged again at 14,000 × *g* for 15 min; the filtrates were collected for peptide quantification (OD280).

Peptides (100 μg) were obtained from each sample and labeled according to the instructions provided by the manufacturer (TMT Mass Tagging Kits and Reagents; Thermo Fisher Scientific, Waltham, MA, United States). The labeled peptides in each group were mixed in equal amount and graded using a high pH reversed-phase spin column. Following lyophilization, peptides (100 μg) were diluted in 0.1% trifluoroacetic acid (T6508; Sigma) (300 µl), transferred to the high pH reversed-phase spin column, and centrifuged to collect the flow-through compositions. After adding 300 μl of pure water, the washed compositions were collected by centrifugation, and gradient elution was performed. Following lyophilization, the samples were re-dissolved in 0.1% formic acid (06450; Fluka, Seelze, Germany) (12 μl), and the concentration of the peptide was determined at OD280.

Each sample was separated by HPLC Easy nLC (Thermo Fisher Scientific) at a nl flow rate. Buffer solution A was 0.1% formic acid aqueous solution, while buffer solution B was 0.1% formic acid acetonitrile aqueous solution (acetonitrile was 84%). The chromatographic column was equilibrated with 95% A solution. The sample was loaded from the automatic injector to the loading column (Acclaim PepMap100, 100 μm × 2 cm, nanoViper C18; Thermo Fisher Scientific) and separated by the analytical column (EASY column, 10 cm, ID75 μm, 3 μm, C18-A2; Thermo Fisher Scientific) at a flow rate of 300 nl/min. For gradient elution, the linear gradient of solution B ranged 0–55% for 0–80 min, 55–100% for 80–85 min, and 100% for 85–90 min.

The samples were separated by chromatography and analyzed using a Q-Exactive mass spectrometer (Thermo Fisher Scientific). The analysis time was 60 min. The detection method was positive ion mode; scanning range of the parent ion was 300–1,800 m/z; resolution of primary MS was 70,000 at 200 m/z; AGC target was 3e6; primary maximum IT was 10 ms; number of scan ranges was 1; and dynamic exclusion was 40.0 s. The mass-to-charge ratios of polypeptide and polypeptide fragments were collected using the following method: 10 MS2 scans were collected after each full scan; the MS2 activation type was higher-energy collisional dissociation; isolation window was 2 m/z; resolution of secondary MS was 35,000 at 200 m/z (TMT6plex, Thermo Fisher Scientific); microscan was 1; secondary maximum IT was 60 ms; normalized collision energy was 30 eV; and underfill was 0.1%.

The raw data of MS were obtained in RAW files, and identified and quantitatively analyzed by Mascot2.2 and Proteome Discoverer 1.4 (Thermo Fisher Scientific). Blast2GO (Götz et al., 2008) was used to perform the Gene Ontology (GO) annotation of the target protein set. The Kyoto Encyclopedia of Genes and Genomes (KEGG) Automatic Annotation Server (Moriya et al., 2007) was used to perform the KEGG pathway annotation of the target protein set. The target protein sequence was classified through KEGG Orthology (KO) by comparison with data obtained from the KEGG GENES database. Information on the pathway involved in the target protein sequence was automatically obtained according to the KO classification. Fisher’s exact test was used to compare the distribution of each GO classification or KEGG pathway in the target protein set, as well as the overall protein set for evaluating the significance level of protein enrichment of a GO term or KEGG pathway. Firstly, the quantitative information of the target protein set was normalized (normalized to the [−1, 1] region). Secondly, the Cluster 3.0 software was used to classify the two dimensions of sample and protein expression (distance algorithm: Euclidean; connection mode: average linkage). Finally, the hierarchical clustering heat map was generated using the Java Treeview software, and the gene symbol of the target protein was obtained from the database of the source of the target protein sequence. These gene symbols were used in the IntAct (http://www.ebi.ac.uk/intact/main.xhtml) or Search Tool for the Retrieval of Interacting Genes (STRING; http://string-db.org/) database to search for direct and indirect interactions between target proteins; the interaction network was generated and analyzed using the Cytoscape 3.2.1 software.

## Results

### High Performance Liquid Chromatography Analysis of Alkaloids Contents in Unprocessed Lateral Root of *Aconitum carmichaelii* and Yinfupian

**FIGURE 3:**
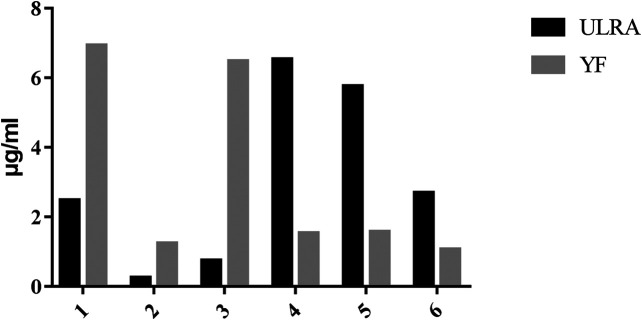
Results of content determination of six alkaloids (*n* = 3). (1), Benzoylmesaconine; (2), Benzoylhypaconine; (3), Benzoylaconine; (4), Mesaconitine; (5), Aconitine; (6), Hypaconitine.

The contents of six alkaloids in ULRA and YF are calculated by the calibration curves (Table 1), and the results are shown in Figure 3. When comparing of ULRA with YF, the contents of aconitine, mesaconitine, and hypaconitine in YF were lower than those of them in ULRA. The contents of benzoylaconine, benzoylmesaconine, and benzoylhypaconine in YF were obvious higher than those of them in ULRA. The diester alkaloids (aconitine, mesaconitine, and hypaconitine) in “Fuzi” were decreased after “Fuzi” processed into YF, and the diester alkaloids may turned into monoester alkaloids, therefore the contents of benzoylaconine, benzoylmesaconine, and benzoylhypaconine in YF are high.

### Analysis of Markers of Differential Components

**FIGURE 4:**
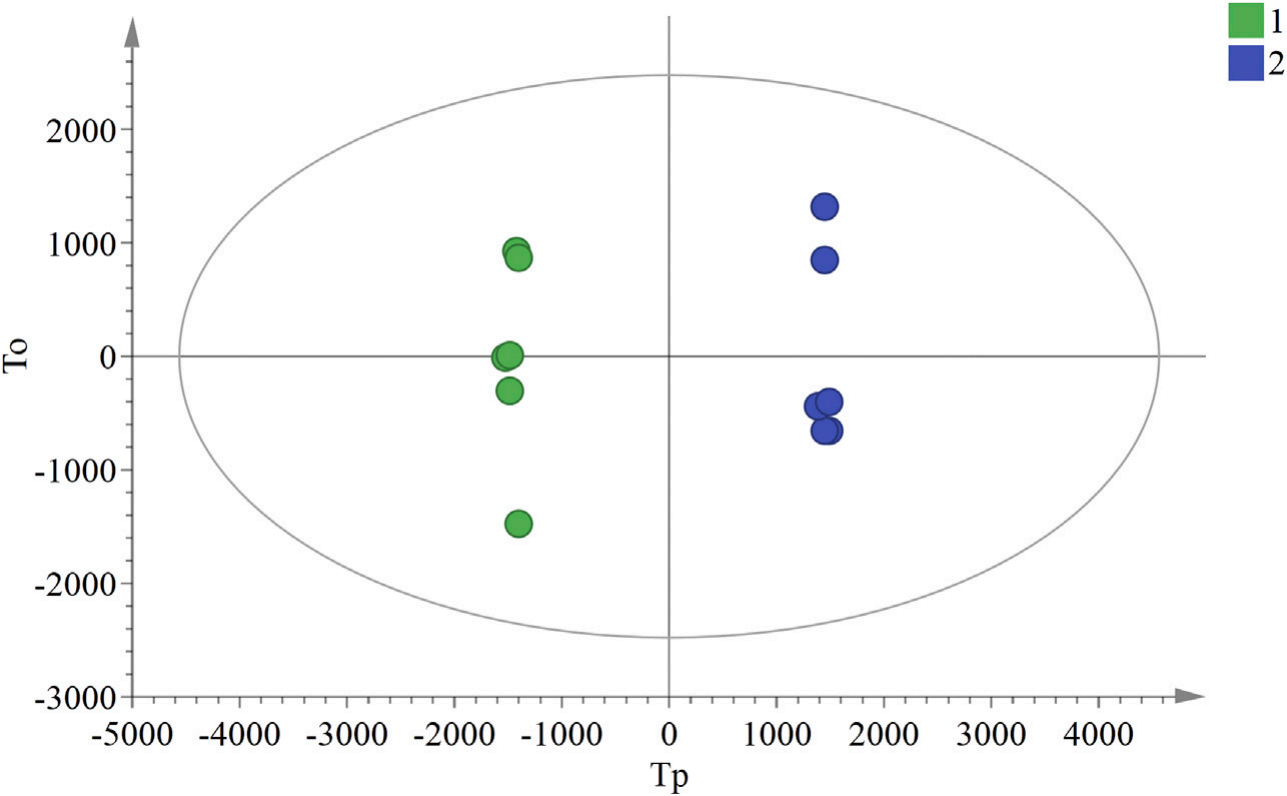
OPLS-DA score chart of ULRA and YF groups. Green dots are ULRA group, and blue dots are YF group. OPLS-DA, partial least squares discrimination analysis; ULRA, unprocessed lateral root of *Aconitum carmichaelii*; YF, Yinfupian.

**FIGURE 5:**
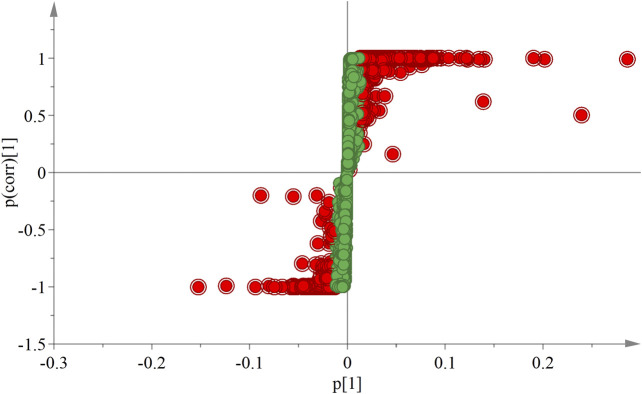
S-plot chart of ULRA and YF groups. The VIP value of red dots is greater than 1. ULRA, unprocessed lateral root of *Aconitum carmichaelii*; YF, Yinfupian; VIP, variable important in projection.

In the OPLS-DA analysis, there were no abnormal samples that were excluded (all samples were within the confidence interval). The score and S-plot chart, which can directly reflect the differences between two groups and the contribution rate of differences in components, were obtained. All the samples of the score chart were clearly distinguished, suggesting that the two groups of samples were significantly different (Figure 4), Two principal components were obtained: R2X = 0.701, R2Y = 0.999, Q2 = 0.991. In the S-plot chart (Figure 5), closer proximity to the two corners of the S-plot indicated a larger VIP value of the variable (i.e., the component with great difference).

According to the data obtained by HPLC-quadrupole-TOF-MS, a comparative analysis was performed between the ULRA and YF groups. The VIP value of the OPLS-DA model principal component (threshold value > 1) and the *p*-value of the *t*-test (threshold: <0.05) were used to screen different components, combined with literature, MassBank database, and other searches. The structure of the differential substances was finally identified as representative of the difference in components (Table 3).

**TABLE 3:** Screening and analysis of the components of ULRA and YF.

No	t_R_/min	Type	Name	Formula	[M + H]^+^ (m/z)	Main fragment ions (m/z)	ULRA/YF	References
1	3.3	C_20_DA	Chuanfumine	C_22_H_35_NO_5_	394.3	376.2479 [M+H-H_2_O]^+^, 358.2385 [M+H-2H_2_O]^+^, 340.2278 [M+H-3H_2_O]+	↓	Liu et al. (2011); Tan et al. (2011); Zhang et al. (2012); Huang et al. (2015)
2	4.5	C_19_ADA	Senbusine A	C_23_H_37_NO_6_	424.3	406.2580 [M+H-H_2_O]^+^, 388.2484 [M+H-2H_2_O]^+^, 374.2337 [M+H-H_2_O-CH_3_OH]^+^, 360.2179 [M+H-2CH_3_OH]^+^, 356.2223 [M+H-2H_2_O-CH_3_OH]^+^	↓	Tan et al. (2011); Huang et al. (2015)
3	4.8	C_19_ADA	Mesaconine	C_24_H_39_NO_9_	486.3	468.2589 [M+H-H_2_O]^+^, 454.2425 [M+H-CH_3_OH]^+^, 436.2312 [M+H-CH_3_OH-H_2_O]^+^, 422.2170 [M+H-2CH_3_OH]^+^, 404.2057 [M+H-2CH_3_OH-H_2_O]^+^, 378.1907 [M+H-C_2_O_2_-3H_2_O]^+^	↓	Tan et al. (2011); Huang et al. (2015)
4	5.3	C_19_ADA	Karakoline	C_22_H_35_NO_4_	378.3	360.2519 [M+H-H_2_O]^+^, 332.2219 [M+H-CH_3_-CH_3_O]^+^, 328.2267 [M+H-CH_3_OH-H_2_O]^+^, 310.2173 [M+H-CH_3_OH-2H_2_O]^+^	↓	Huang et al. (2015)
5	5.4	C_19_ADA	Isotalatizidine	C_23_H_37_NO_5_	408.3	390.2641 [M+H-H_2_O]^+^, 372.2550 [M+H-2H_2_O]^+^, 358.2389 [M+H-H_2_O-CH_3_OH]^+^	↓	Tan et al. (2011); Huang et al. (2015)
6	5.8	C_20_DA	Songorine	C_22_H_31_NO_3_	358.2	340.2266 [M+H-H_2_O]^+^, 322.2178 [M+H-2H_2_O]^+^	↓	Tan et al. (2011); Zhang et al. (2012); Huang et al. (2015)
7	5.9	C_19_ADA	Aconine	C_25_H_41_NO_9_	500.3	482.2748 [M+H-H_2_O]^+^, 468.2592 [M+H-CH_3_OH]^+^, 450.2479 [M+H-CH_3_OH-H_2_O]^+^, 436.2335 [M+H-2CH_3_OH]^+^, 418.2229 [M+H-2CH_3_OH-H_2_O]^+^	↓	Huang et al. (2015)
8	7.0	C_19_ADA	Fuziline	C_24_H_39_NO_7_	454.3	436.2669 [M+H-H_2_O]^+^, 418.2591 [M+H-2H_2_O]^+^, 404.2420 [M+H-CH_3_OH-H_2_O]^+^, 386.2327 [M+H-CH_3_OH-2H_2_O]^+^, 354.2066 [M+H-2CH_3_OH-2H_2_O]^+^	↓	Tan et al. (2011); Sun et al. (2012); Huang et al. (2015)
9	7.0	C_19_ADA	Hypaconine	C_24_H_39_NO_8_	470.3	438.2470 [M+H-CH_3_OH]^+^, 406.2217 [M+H-2CH_3_OH]^+^, 388.2114 [M+H-2CH_3_OH-H_2_O]^+^, 378.1903 [M+H-2CH_2_O-CH_3_OH]^+^, 374.1961 [M+H-3CH_3_OH]^+^, 356.1853 [M+H-3CH_3_OH-H_2_O]^+^	↓	Huang et al. (2015)
10	8.0	C_19_ADA	Neoline	C_24_H_39_NO_6_	438.3	420.2737 [M+H-H_2_O]^+^, 402.2648 [M+H-2H_2_O]^+^, 388.2474 [M+H-H_2_O-CH_3_OH]^+^, 374.2341 [M+H-2CH_3_OH]^+^, 370.2382 [M+H-2H_2_O-CH_3_OH]^+^, 356.2220 [M+H-2CH_3_OH-H_2_O]^+^	↓	Liu et al. (2011); Tan et al. (2011); Huang et al. (2015)
11	8.8	C_19_ADA	Talatizamine	C_24_H_39_NO_5_	422.3	390.2644 [M+H-CH_3_OH]^+^, 372.2546 [M+H-CH_3_OH-H_2_O]^+^, 358.2395 [M+H-2CH_3_OH]^+^, 340.2289 [M+H-2CH_3_OH-H_2_O]^+^	↓	Tan et al. (2011); Sun et al. (2012); Huang et al. (2015)
12	11.7	C_19_MDA	14-Benzoyl-10-OH−mesaconine	C_31_H_43_NO_11_	606.3	588.2788 [M+H-H_2_O]^+^, 574.2636 [M+H-CH_3_OH]^+^, 556.2515 [M+H-CH_3_OH-H_2_O]^+^, 542.2376 [M+H-2CH_3_OH]^+^, 524.2262 [M+H-2CH_3_OH-H_2_O]^+^	↓	Tan et al. (2011)
13	14.3	C_19_MDA	Benzoylmesaconine	C_31_H_43_NO_10_	590.3	558.2683 [M+H-CH_3_OH]^+^, 540.2571 [M+H-CH_3_OH-H_2_O]^+^, 526.2430 [M+H-2CH_3_OH]^+^, 508.2316 [M+H-2CH_3_OH-H_2_O]^+^	↓	Tan et al. (2011); Huang et al. (2015)
14	15.6	C_19_MDA	Benzoylaconine	C_32_H_45_NO_10_	604.3	586.3005 [M+H-H_2_O]^+^, 572.2836 [M+H-CH_3_OH]^+^, 540.2591 [M+H-2CH_3_OH]^+^, 554.2726 [M+H-CH_3_OH-H_2_O]^+^, 522.2477 [M+H-2CH_3_OH -H_2_O]^+^	↓	Tan et al. (2011); Huang et al. (2015)
15	16.5	C_19_MDA	Benzoylhypaconine	C_31_H_43_NO_9_	574.3	542.2733 [M+H-CH_3_OH]^+^, 524.2661 [M+H-CH_3_OH-H_2_O]^+^, 510.2482 [M+H-2CH_3_OH]^+^, 492.2391 [M+H-2CH_3_OH-H_2_O]^+^	↓	Tan et al. (2011); Huang et al. (2015)
16	17.7	C_19_MDA	14-Benzoyl-13-deoxyhyaconine	C_31_H_43_NO_8_	558.3	526.2807 [M+H-CH_3_OH]^+^, 508.2718 [M+H-CH_3_OH-H_2_O]^+^, 494.2548 [M+H-2CH_3_OH]^+^, 476.2449 [M+H-2CH_3_OH-H_2_O]^+^	↓	Tan et al. (2011)
17	17.9	C_19_MDA	Benzoyldeoxyaconine	C_32_H_45_NO_9_	588.3	556.2900 [M+H-CH_3_OH]^+^, 538.2832 [M+H-CH_3_OH-H_2_O]^+^, 524.2650 [M+H-2CH_3_OH]^+^, 496.2347 [M+H-2CH_3_OH-H_2_O]^+^	↓	Tan et al. (2011); Huang et al. (2015)
18	17.9	C_19_DDA	Beiwutine	C_33_H_45_NO_12_	648.3	616.2781 [M+H-CH_3_OH]^+^, 598.2683 [M+H-CH_3_OH-H_2_O]^+^, 588.2822 [M+H-2CH_2_O]^+^, 556.2563 [M+H-2CH_2_O-CH_3_OH]^+^, 538.2457 [M+H-2CH_2_O-CH_3_OH-H_2_O]^+^	↑	Huang et al. (2015)
19	19.4	C_19_MDA	Dehydrated-benzoylhypaconine	C_31_H_41_NO_8_	556.3	524.2641 [M+H-CH_3_OH]^+^, 492.2391 [M+H-2CH_3_OH]^+^	↓	Huang et al. (2015)
20	19.7	C_19_DDA	Mesaconitine	C_33_H_45_NO_11_	632.3	600.2823 [M+H-CH_3_OH]^+^, 582.2740 [M+H-CH_3_OH-H_2_O]^+^, 572.2874 [M+H-2CH_2_O]^+^, 540.2626 [M+H-2CH_2_O-CH_3_OH]^+^	↑	Sun et al. (2012); Huang et al. (2015)
21	21.3	C_19_DDA	Hypaconitine	C_33_H_45_NO_10_	616.3	584.2844 [M+H−CH_3_OH]^+^, 556.2879 [M+H-2CH_2_O]^+^, 524.2621 [M+H-2CH_2_O-CH_3_OH]^+^, 492.2373 [M+H-C_2_O_2_-CH_3_OH-2H_2_O]^+^	↑	Tan et al. (2011); Huang et al. (2015)
22	21.5	C_19_DDA	Aconitine	C_34_H_47_NO_11_	646.3	628.3069 [M+H-H_2_O]^+^, 596.2855 [M+H-CH_3_OH-H_2_O]^+^, 586.3053 [M+H−AcOH]^+^, 568.2895 [M+H−AcOH−H_2_O]^+^, 554.2785 [M+H−AcOH−CH_3_OH]^+^, 536.2693 [M+H-2CH_2_O-CH_3_OH-H_2_O]^+^	↑	Tan et al. (2011); Sun et al. (2012); Huang et al. (2015)
23	23.3	C_19_DDA	Deoxyaconitine	C_34_H_47_NO_10_	630.3	598.3029 [M+H−CH_3_OH]^+^, 570.3061 [M+H−AcOH]^+^, 538.2804 [M+H−AcOH−CH_3_OH]^+^, 506.2549 [M+H−AcOH−2CH_3_OH]^+^	↑	Tan et al. (2011); Huang et al. (2015)

Note: ULRA compared with YF, ↑, the content increased; ↓, the content decreased; C_19_ADA, C19-alkylol amine-diterpenoid alkaloid; C_19_DDA, C19-monoester-diterpenoid alkaloid; C_19_MDA, C19-monoester-diterpenoid alkaloid; C_20_DA, C20-diterpene alkaloid; ULRA, unprocessed lateral root of *Aconitum carmichaelii*; YF, Yinfupian.

Identification of components: The inference process was illustrated using chuanfumine as an example. Compound 1 produced 394.3 [M+H]^+^ with high sensitivity at 40 eV collision energy. In the MS2 spectrum, the peak produced 376.2479 [M+H-H_2_O]^+^, 358.2385 [M+H-2H_2_O]^+^, and 340.2278 [M+H-3H_2_O]^+^, according to the literature (Liu et al., 2011; Zhang et al., 2012). There was no [M+H-CH_3_OH-H_2_O]^+^, which is a characteristic ion of karakolidine (C_22_H_35_NO_5_). Therefore, it was inferred that compound 1 was chuanfumine.

### Histomorphological Changes in the Gonads of Rats Induced by Yang Deficiency Syndrome

**FIGURE 6:**
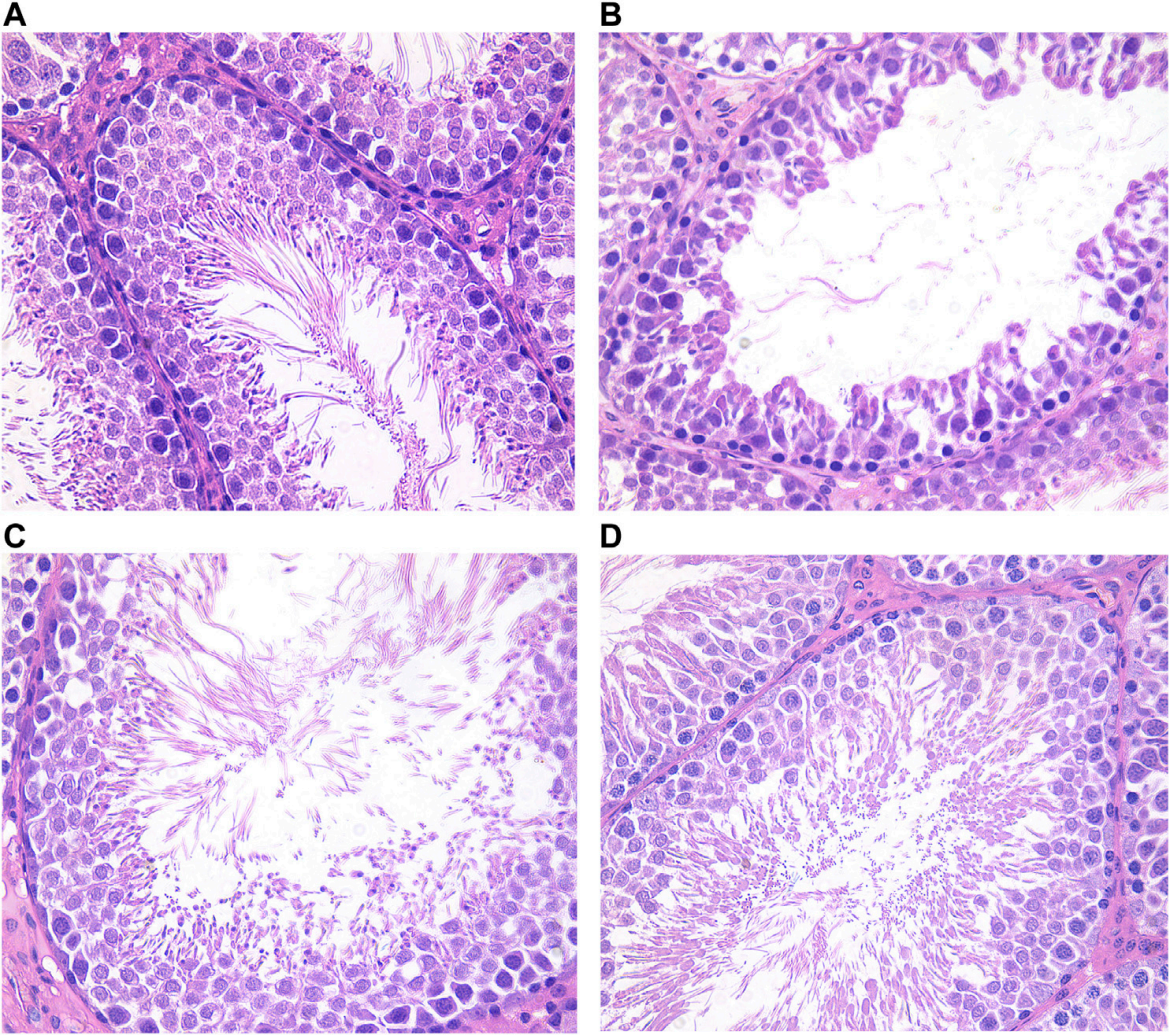
Testis histological changes of male rats in each group (HE×400). **(A)** Blank group; **(B)** Model group; **(C)** ULRA group; **(D)** YF group. HE, hematoxylin-eosin staining; ULRA, unprocessed lateral root of *Aconitum carmichaelii*; YF, Yinfupian.

Ovarian and testicular tissues were obtained from female and male rats, respectively. In the blank group, the histological structure of the testes was normal, and the division of spermatogenic cells in the seminiferous tubules was active with rich and obvious layers (Figure 6A). In the model group, the number of spermatogenic cells and sperm cells was significantly lower, and the arrangement was loose with vacuoles (Figure 6B).

Compared with the model group, the number of spermatogenic cells and sperm cells in the ULRA and YF groups was significantly increased and orderly arranged, similar to the blank group, and recovered significantly (Figures 6C,D).

**FIGURE 7:**
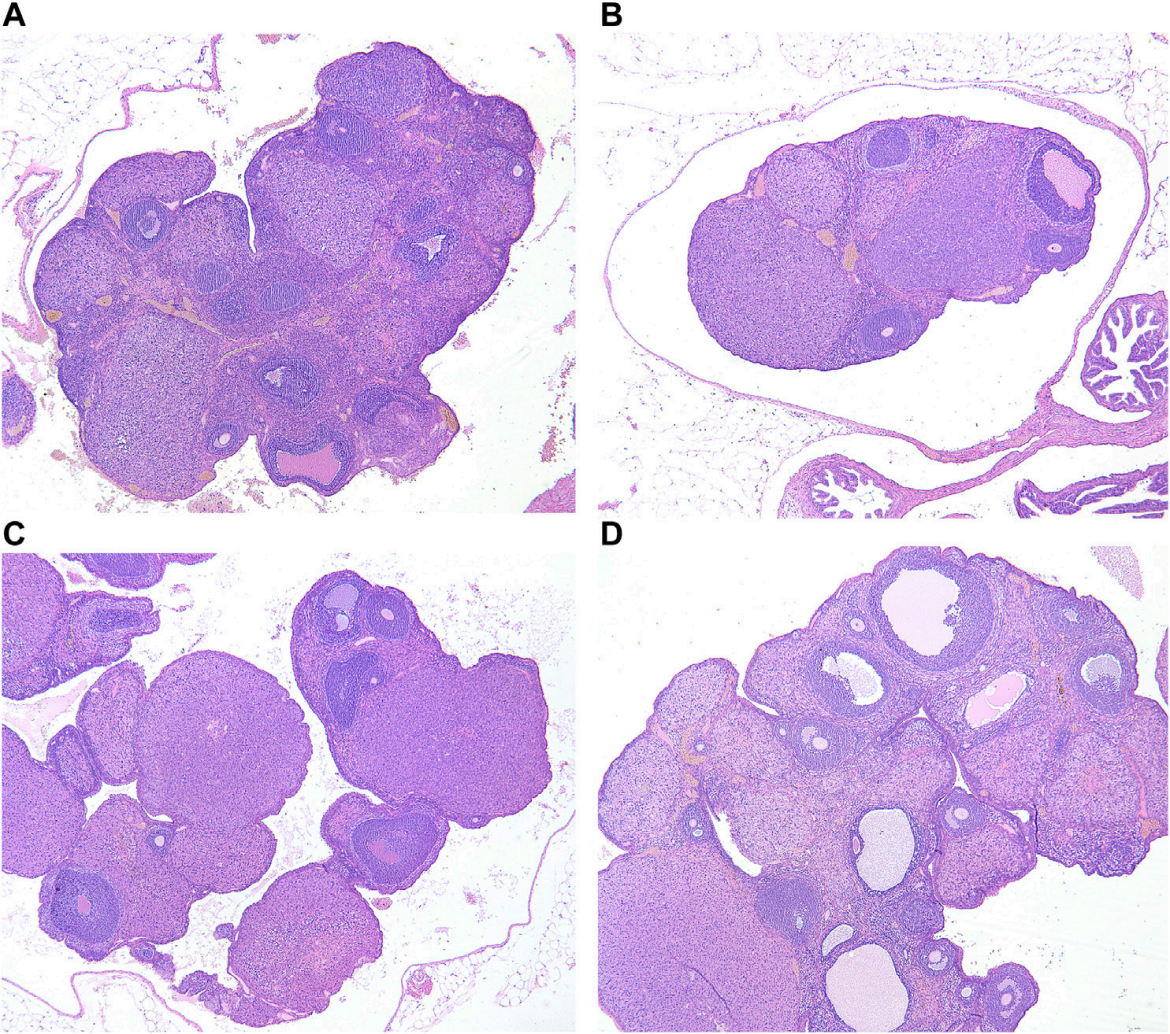
Ovarian histological changes of female rats in each group (HE×40). **(A)** Blank group; **(B)** Model group; **(C)** ULRA group; **(D)** YF group; HE, hematoxylin-eosin staining; ULRA, unprocessed lateral root of *Aconitum carmichaelii*; YF, Yinfupian.

In the blank group, the number and morphology of ovarian follicles at all levels were normal; the ovarian follicles grew actively, and there were abundant layers of granulosa cells (Figure 7A). In the model group, the volume of the ovary decreased, and the number of ovarian follicles and granulosa cells in different layers decreased significantly (Figure 7B).

Compared with the model group, the ovarian volume and the number of granulosa cells in the ULRA group increased (Figure 7C). The volume of ovary and the number of granulosa cells, corpus luteum, and ovarian follicles increased in the YF group (Figure 7D).

### Serum cyclic Adenosine Monophosphate and cyclic Guanosine Monophosphate Levels in Rats with Yang Deficiency Syndrome

Compared with the blank group, the levels of c-AMP and c-GMP in the model group were significantly decreased. There was no significant difference between the ULRA and model groups. Compared with the model group, the levels of c-AMP in the YF group increased significantly, whereas those of c-GMP exhibited an upward trend.

### Differentially Expressed Proteins

According to the standard of expression <1.2-fold (upregulated by >1.2-fold or downregulated by <0.83-fold) and *p*-value <0.05, a total of 4,673 differentially expressed proteins were identified. The number of differentially expressed proteins in each group is shown in Table 4.

**TABLE 4:** Protein expression results.

Comparison (group)	Upregulation	Downregulation	All
B vs. M	30	35	65
ULRA vs. M	27	135	162
YF vs. M	52	46	98

Note: Upregulation, upregulated differentially expressed proteins; Downregulation, downregulated differentially expressed proteins; All, all differentially expressed proteins; B, blank group; M, model group; ULRA, unprocessed lateral root of *Aconitum carmichaelii*; YF, Yinfupian.

### Gene Ontology Analysis

**FIGURE 8:**
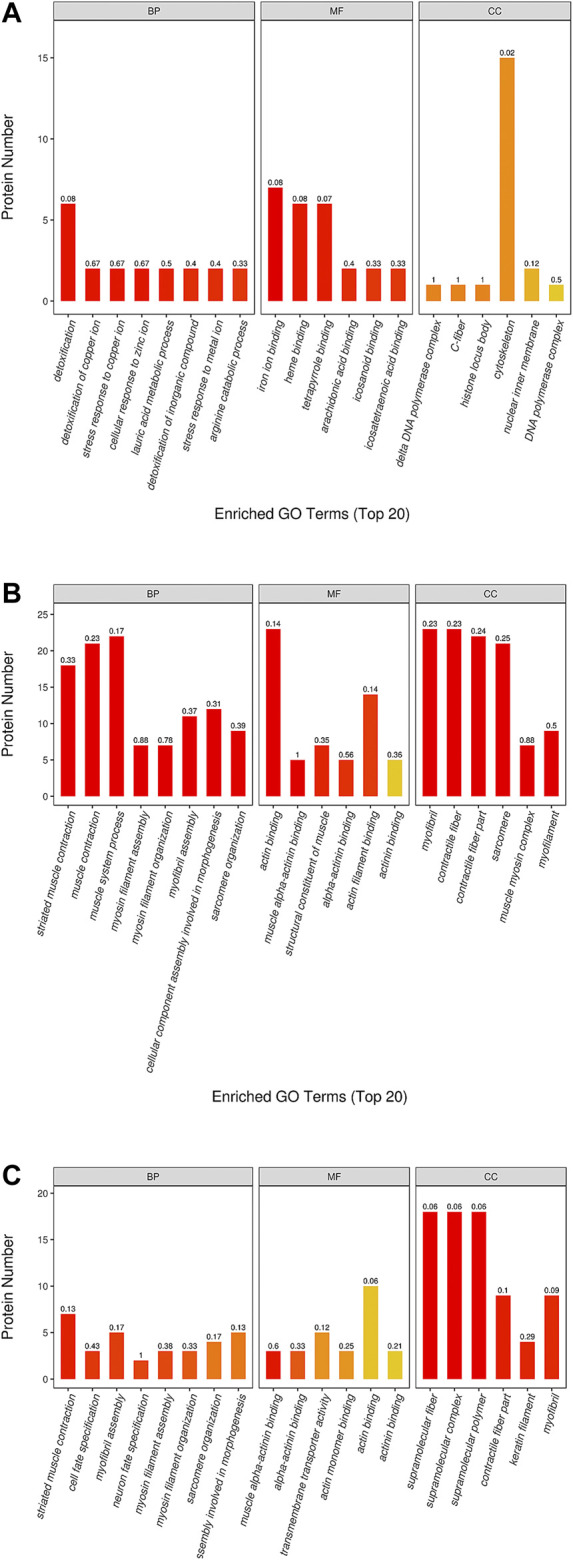
The GO (gene ontology) function classification of the differential protein identified by the TMT-based proteomics. The GO enrichment analysis of proteins from the liver samples was performed according to the BP (biological process), MF (molecular function) and CC (cellular component). The comparison between the **(A)** blank group and model group, between the **(B)** ULRA group and model group, between the **(C)** YF group and model group was performed. ULRA, unprocessed lateral root of *Aconitum carmichaelii*; YF, Yinfupian.

The results of GO analysis for the blank group vs. the model group revealed that the functions of these potential targets are related to numerous biological processes that may be important for the occurrence and development of YDS. The top 20 generally changed GO terms were compared. The most significantly enriched biological process terms were associated with detoxification, stress response to metal ion, cellular response to zinc ion, lauric acid metabolic process, and arginine catabolic process. With respect to molecular function, the most significantly enriched terms included iron ion binding, heme binding, tetrapyrrole binding, arachidonic acid binding, icosanoid binding, and icosatetraenoic acid binding. For the cellular component, the most enriched terms were delta DNA polymerase complex, C-fiber, histone locus body, cytoskeleton, nuclear inner membrane, and DNA polymerase complex (Figure 8A).

Comparison of the ULRA and model groups showed that the most enriched biological process terms were associated with muscle contraction, muscle system process, myosin filament assembly and organization, myofibril assembly, sarcomere organization, and cellular component assembly involved in morphogenesis. The representative terms related to molecular function were actin binding and structural constituent of muscle. For the cellular component, the most enriched terms were myofibril, contractile fiber, sarcomere, and muscle myosin complex (Figure 8B).

In the YF group vs. model group analysis, the most significantly enriched biological process terms were associated with muscle contraction, cell fate specification, myosin filament assembly and organization, sarcomere organization, and cellular component assembly involved in morphogenesis. The most significantly enriched molecular function terms were associated with actin binding and transmembrane transporter activity. The representative terms related to cellular component were supramolecular structure, contractile fiber part, keratin filament, and myofibril (Figure 8C).

### Kyoto Encyclopedia of Genes and Genomes Analysis

**FIGURE 9:**
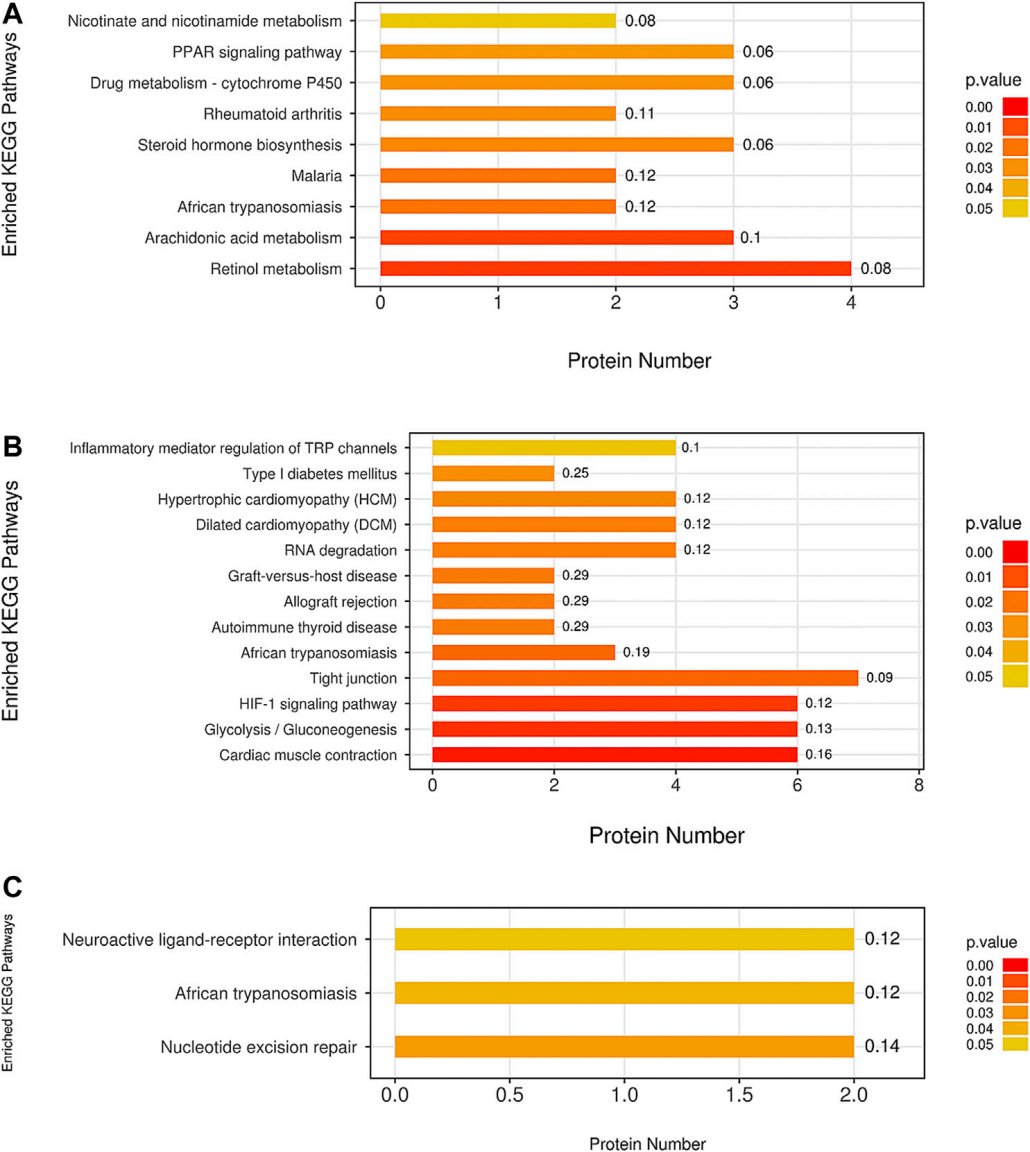
The pathway analysis of all differentially expressed proteins was based on the KEGG (Kyoto Encyclopedia of Genes and Genomes) database. The comparison between the **(A)** M group and B group, between the **(B)** ULRA group and model group, and between the **(C)** YF and model groups was performed. ULRA, unprocessed lateral root of *Aconitum carmichaelii*; YF, Yinfupian.

The KEGG analysis demonstrated that a number of pathways were significantly enriched in the model group compared with the blank group. These included some classical pathways, such as the nicotinate and nicotinamide metabolism, peroxisome proliferator activated receptor (PPAR) signaling pathway, drug metabolism cytochrome P450 (CYP), steroid hormone biosynthesis, arachidonic acid metabolism, and retinol metabolism (Figure 9A).

Comparison of the ULRA and model groups revealed that the inflammatory mediator regulation of transient receptor potential (TRP) channels, hypoxia inducible factor 1 (HIF1) signaling pathway, tight junction, glycolysis/gluconeogenesis, and cardiac muscle contraction were significantly enriched according to the differentially expressed proteins. Moreover, we found some disease-related pathways, such as type I diabetes mellitus, hypertrophic cardiomyopathy, dilated cardiomyopathy, allograft rejection, graft-versus-host disease, autoimmune thyroid disease, and African trypanosomiasis, which indicate that raw aconite has a potential application in other diseases (Figure 9B).

Comparison of the YF and model groups showed neuroactive ligand-receptor interaction and nucleotide excision repair (Figure 9C).

### Protein–Protein Interaction

To investigate the interactions between differentially expressed proteins in each group, several protein–protein interaction networks were established based on differentially expressed proteins using the Cytoscape v3.2.1 software. Comparison of the model and blank groups revealed complex interactions with CYP 4A2 and 4A8 (downregulation), aldehyde oxidase 3 (AOX3) (downregulation), cathepsin G (CTSG) (upregulation), fatty acid binding protein 5 (FABP5) (upregulation), lysozyme 2 (LYZ2) (upregulation), etc. In the comparison of the ULRA and model groups, the protein network based on differentially expressed proteins revealed complex interactions with actinin alpha 3 (ACTN3) (downregulation), actinin alpha 2 (ACTN2) (downregulation), troponin C2 fast skeletal type (TNNC2) (downregulation), troponin I2 fast skeletal type (TNNI2) (downregulation), troponin T3 fast skeletal type (TNNT3) (downregulation), creatine kinase M-type (CKM) (downregulation), myosin light chain 1 (MYL1) (downregulation), myosin heavy chain 1 (MYH1) (downregulation), etc. Comparison of the YF and model groups showed complex interactions with keratin 5 (KRT5) (upregulation), KRT16 (upregulation), KRT17 (upregulation), KRT72 (upregulation), mitochondrial ribosomal protein L28 (MRPL28) (upregulation), MRPL4 (upregulation), MRPL38 (upregulation), OXA1L mitochondrial inner membrane protein (OXA1L) (upregulation), etc.

## Discussion

*Aconitum* alkaloids can be rapidly absorbed and widely distributed in the body. There is a large quantity of C19-diester-diterpenoid alkaloid (C_19_DDA) in ULRA; C_19_DDA has been associated with acute toxicity. Hydrolysis of C_19_DDA into C19-monoester-diterpenoid alkaloid (C_19_MDA) decreases the toxicity. Through further hydrolysis, the alkaloids are converted into C19-alkylol amine-diterpenoid alkaloid (C_19_ADA) with almost no toxicity (Huang et al., 2015; Wu et al., 2018). According to the results of composition analysis (Table 1), the levels of mesaconitine, hypaconitine, aconitine, and other C_19_DDAs were significantly decreased in YF compared with the ULRA. In contrast, the levels of C_19_MDAs (e.g., benzoylaconine, benzoylhypaconine, benzoylmesaconine), C_19_ADAs (e.g., fuziline, mesaconine, and neoline), and C20-diterpene alkaloids (e.g., chuanfumine and songorine) were significantly increased. The components of *Aconitum* alkaloids underwent obvious changes in the process of preparation. Hydrolysis of C_19_DDA reduced the toxicity of ULRA, while the increase in the levels of C_19_MDA, C_19_ADA, and C20-diterpene alkaloid also ensured the pharmacological activity. This confirmed the importance of preparation in reducing the toxicity and increasing efficiency of ULRA in terms of chemical composition.

**FIGURE 10:**
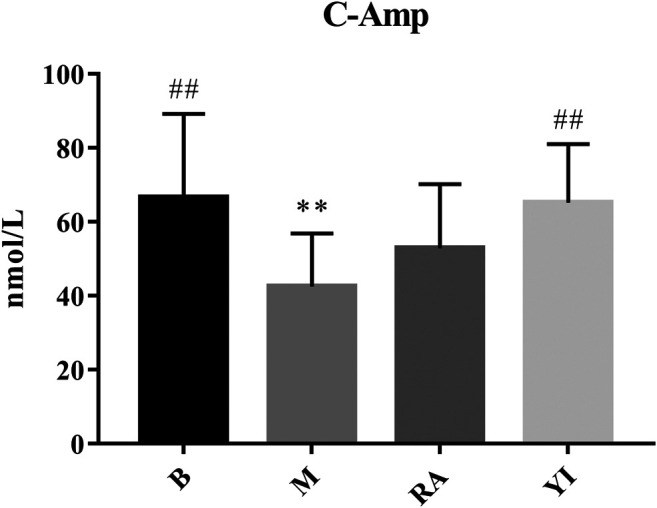
Changes of serum c-AMP levels. B, blank group; M, model group; RA, ULRA group; YI, YF group. Compared with B group, **: *p* < 0.01; Compared with M group, ##: *p* < 0.01. c-AMP, cyclic adenosine monophosphate; ULRA, unprocessed lateral root of *Aconitum carmichaelii*; YF, Yinfupian.

**FIGURE 11:**
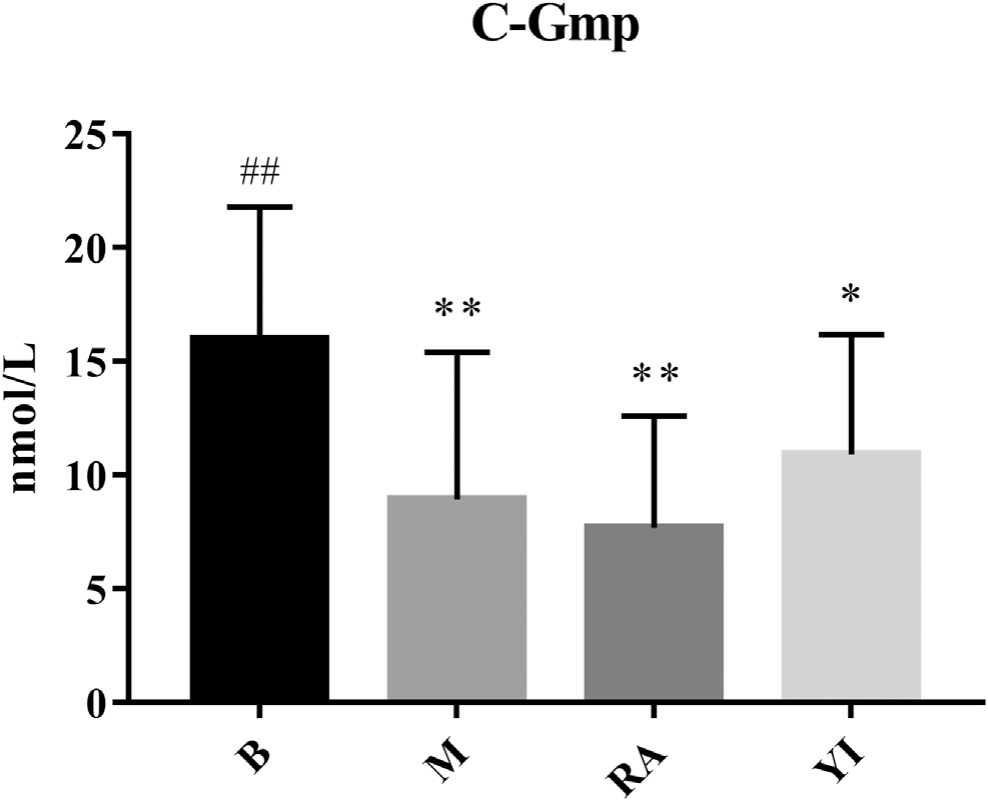
Changes of serum c-GMP levels. B, blank group; M, model group; RA, ULRA group; YI, YF group. Compared with B group, *: *p* < 0.05, **: *p* < 0.01; Compared with M group, ##: *p* < 0.01. c-GMP, cyclic guanine monophosphate; ULRA, unprocessed lateral root of *Aconitum carmichaelii*; YF, Yinfupian.

Previous studies have shown that YDS can affect gonad and related functions, as well as damage male testicular function and female ovarian function (Nakada and Adachi. 1999; Liu et al., 2018; Song et al., 2019). The vitality of spermatogenic cells and ovarian cells in the testes and ovaries of rats with YDS was inhibited, and the numbers of cells were significantly reduced (Figures 3, 4). Moreover, following treatment with ULRA and YF, the morphology of the testes and ovaries recovered well in rats with YDS, suggesting good efficacy.

c-AMP is a signal transduction substance formed by the dephosphorylation of adenosine triphosphate (ATP) under the catalysis of adenylate cyclase It activates protein kinase (PKA) and phosphorylates target cell proteins, thus regulating the cell response. c-GMP is another signal transduction substance formed by guanosine triphosphate under the catalysis of guanylate cyclase. It activates protein kinase PKG and phosphorylates proteins in target cells, thus regulating cell reaction. Both transduction substances, termed cyclic nucleotides, are involved in regulating the physiological function and metabolism of cells. They have a wide range of biological effects and participate in a variety of physiological and pathological processes. During this period, numerous neurotransmitters, hormones and some active substances need to exert their corresponding physiological effects on target cells through cyclic nucleotides (Burhenne et al., 2011; Rondina and Weyrich, 2012). The content of c-AMP and c-GMP in rats with YDS decreased significantly, thereby leading to the inhibition of physiological function and metabolism to a certain extent. The body reaction caused by this change was consistent with the symptoms of YDS, and YF could significantly increase the content of c-AMP and c-GMP (Figures 10, 11). The above results indicate that YF has an obvious therapeutic effect on YDS.

The GO and KEGG pathway enrichment analyses revealed that YDS mainly affects detoxification, heme-related compositions (i.e., iron ion, heme, tetrapyrrole) binding, response to metal ion, CYP, steroid hormone biosynthesis, nicotinate and nicotinamide metabolism, PPAR signaling pathway, as well as the combination and metabolism of some unsaturated fatty acids. The present findings are consistent with those of previous research studies on YDS (Zhang et al., 2019; Liang et al., 2020). GO analysis showed that both ULRA and YF influenced the movement of muscle tissues. KEGG pathway analysis found that ULRA mainly affects the inflammatory mediator regulation of TRP channels, HIF1 signaling pathway, tight junction, glycolysis/gluconeogenesis, and cardiac muscle contraction, and was related to some disease-related pathways, such as type I diabetes mellitus, hypertrophic cardiomyopathy, dilated cardiomyopathy, allograft rejection, graft-versus-host disease, autoimmune thyroid disease, and African trypanosomiasis. YF was mainly associated with neuroactive ligand-receptor interaction and nucleotide excision repair. The above results may be closely related to the content of alkaloids in RAC. The high content of C_19_DDA in ULRA has been linked to strong anti-inflammatory activity (Hikino et al., 1982; Komoda et al., 2003), anti-tumor activity (Du et al., 2013; Ji et al., 2016), and damage to myocardial cells (Ma et al., 2018). The high content of C_19_MDA in YF exerts a protective effect on nerve cells (Jiang et al., 2012). C_19_ADA can increase the viability of myocardial cells, and improve myodynamia and ventricular diastolic function (Liu et al., 2012; Xiong et al., 2012).

CYP has two main biological functions. The first is the metabolism of heterologous substances. Lipid soluble drugs can only be excreted after biotransformation in the kidneys (Modi and Dawson, 2015). CYP can reduce the hydrophobicity of compounds and form intermediate metabolites for easy excretion. The second function is the biosynthesis of bioactive molecules, including the metabolism of steroids, vitamins, and fatty acids (Albertolle and Guengerich, 2018). The CYP4A family metabolizes arachidonic acid into ω-hydroxyeicosatetraenoic acid in kidneys (Ying et al., 2008). 20-hydroxyeicosatetraenoic acid plays an important role in skeletal muscle and vascular myogenic response, and regulates blood circulation (Imig et al., 1996). In addition, studies have shown that specific inhibition of CYP4A can treat myocardial injury induced by advanced glycation end product (Wang et al., 2019). Aldehyde oxidase is an important enzyme system involved in drug metabolism (Garattini et al., 2003). *In vivo* studies showed that CTSG inhibitors decreased cardiac inflammation and improved cardiac function after myocardial ischemia-reperfusion injury (Hooshdaran et al., 2017), indicating that CTSG may promote inflammation. FABP is a sensitive marker for the early diagnosis of myocardial infarction (Mad et al., 2007; Figiel et al., 2008). Following the occurrence of myocardial ischemia, fatty acids are mobilized to supply energy, thereby increasing the levels of heart-type FABP. LYZ is widely distributed in the human body because of its antibacterial, antiviral, anti-inflammatory, and immunity-enhancing properties (Eichenberger et al., 2010).

The downregulation of CYP and AOX in the model group may indicate that YDS can inhibit the metabolic activities in the body. Upregulation of CTSG expression can aggravate the inflammatory reaction. The upregulation of FABP and LYZ may be related to the self-regulation and response of the body in the state of YDS. CYP was downregulated in the ULRA group compared with the model group. This finding was consistent with those of previous studies stating that C_19_DDA can inhibit the activity of CYP and affect its mRNA levels. Its toxic targets are ion channels, substructures, enzymes, and receptors (Yen and Ewald, 2012; Chang et al., 2019). The expression of AOX3 was significantly increased in the ULRA group compared with the model group. The levels of CTSG were significantly decreased in the YF group compared with the model group, suggesting that YF may exert an anti-inflammatory effect by downregulating the expression of CTSG.

Together, α-actinin and actin form the main cytoskeleton protein of cells, maintain the special morphology of cells, and endow the cells with toughness and strength (Dixson et al., 2003). For myocardial cells, actinin is located on the myocardial cell membrane and Z-band through a variety of actin connexins to perform myocardial contractile and diastolic functions. α-actinin is one of the connexins; following change, α-actinin directly affects the systolic and diastolic functions of myocardial cells and leads to myocardial remodeling. Spatial regularity disorder of α-actinin-2 has been found in cardiac myocytes of patients with heart failure (Dobrev and Nattel, 2010), indicating that the plasticity of α-actinin-2 may occur in cardiac structural remodeling. TNNT contains the binding site of protomyosin; TNNI is an inhibitor of actin ATPase, which inhibits the interaction between actin and myosin. TNNC binds to Ca^2+^ and regulates the interaction between TNNT, TNNI, and other components of the systolic system (Perry, 1998; Perry, 1999). Creatine kinase (CK) is closely related to energy metabolism in mammals. It is involved in glycolysis control, mitochondrial respiration, and energy supply for muscle contraction. CK is one of the key enzymes in the metabolism of the ATP-creatine phosphate system. Its role is to catalyze the reversible transfer of a high-energy bond between ATP and creatine phosphate (Cheng et al., 2020). Myosin is the structural protein and the main contractile protein of the myocardium. It is composed of two heavy chains and four light chains. Typically, the body can produce immune tolerance to myosin. However, under pathological conditions, myosin can be an autoantigen causing an autoimmune reaction, stimulating the production of anti-myosin antibody, and mediating myocardial immune injury. Studies (Wang et al., 2003) suggested that myosin can be an autoantigen mediating myocardial injury, which can transform myocarditis into dilated cardiomyopathy.

Studies have shown that aconitine can inhibit the expression of α-actinin (Zhang et al., 2020). This may be due to the effects of aconitine on protein expression in the myofilaments of cardiomyocytes, resulting in dysfunction of myofilaments; this may be the mechanism of aconitine-induced cardiotoxicity. The expression levels of troponin and CK were downregulated in the ULRA group, indicating that ULRA could inhibit the contraction and relaxation of muscle. Moreover, the downregulation of myosin may be involved in the anti-inflammatory effect of ULRA.

Mammalian mitochondrial ribosomal proteins are synthesized in cytoplasmic ribosomes and transported to mitochondria by special protein complexes. They are combined with rRNA and assembled into ribosomes. Moreover, they are responsible for the translation of 13 membrane proteins encoded by mitochondrial DNA and participate in the oxidative phosphorylation reaction (Christian and Spremulli, 2011; De et al., 2015). Mitochondrial inner membrane protein is essential for the activity and assembly of cytochrome oxidase; it is also necessary for the correct biogenesis of ATP synthase and complex I in mitochondria (Bonnefoy et al., 1994; Stiburek et al., 2007).

In the YF group, the expression of Mammalian mitochondrial ribosomal proteins was significantly upregulated. Studies have shown that benzoylaconine can increase the mitochondrial quality and induce mitochondrial biogenesis in mice by activating the adenosine monophosphate-activated protein kinase (AMPK) signaling cascade (Deng et al., 2019). It is suggested that YF can promote the production of ATP, and the increase in benzoylaconine is an important step for mitochondrial energy metabolism.

As a medicinal plant with documented efficacy and toxicity, the chemical composition and effects of LRA have been investigated in recent years, shedding light on its material basis and the pharmacological and toxicological mechanisms. However, further studies are warranted to overcome some current limitations. Regarding chemical constituents, despite the availability of detailed studies on various alkaloids, the extraction of many compounds remains difficult, and there are challenges in the hydrolysis and transformation of C_19_DDA, C_19_MDA, and C_19_ADA. Therefore, it is necessary to further investigate the dynamic transformation law of different types of alkaloids to increase efficiency and reduce toxicity in the process of component transformation. Concerning medicinal activity, although the overall pharmacological and toxicological effects of LRA have been extensively studied, there is a lack research on the target mechanism of most monomer compounds. Hence, it is necessary to further clarify the mechanisms of interaction between multiple components and multiple targets.

## Data Availability

The datasets presented in this study can be found in online repositories. The names of the repository/repositories and accession number(s) can be found at [iProX (www.iprox.org), ID: PXD026321].
